# Congenital self-healing reticulohistiocytosis in a newborn: unusual oral and cutaneous manifestations

**DOI:** 10.1186/s13052-021-01082-9

**Published:** 2021-06-10

**Authors:** Alessandra Rizzoli, Simona Giancristoforo, Cristina Haass, Rita De Vito, Stefania Gaspari, Eleonora Scapillati, Andrea Diociaiuti, May El Hachem

**Affiliations:** 1 Neonatal Intensive Unit, San Pietro - Fatebenefratelli Hospital, Rome, Italy; 2 Dermatology Unit, Bambino Gesù Children’s Hospital, IRCCS, Piazza S. Onofrio, 4, 00165 Rome, Italy; 3 Pathology Unit, Bambino Gesù Children’s Hospital, IRCCS, Rome, Italy; 4 Department of Hematology-Oncology and Cellular and Gene Therapy, Bambino Gesù Children’s Hospital, IRCCS, Rome, Italy

**Keywords:** Congenital self-healing reticulohistiocytosis, CSHRH, Hashimoto-Pritzker disease, Histiocytosis, Newborn

## Abstract

**Background:**

Congenital self-healing reticulohistiocytosis (CSHRH), also called Hashimoto-Pritzker disease, is a rare and benign variant of Langerhans cell histiocytosis, characterized by cutaneous lesions without extracutaneous involvement.

**Case presentation:**

We present a case of CSHRH with diffuse skin lesions and erosions in the oral mucosa, present since birth and lasting for 2 months, and we perform a review of the literature on Pubmed in the last 10 years.

**Conclusions:**

Our case confirm that lesions on oral mucosa, actually underestimated, may be present in patients with CSHRH. Patients affected by CSHRH require a close follow-up until the first years of life, due to the unpredictable course of Langerhans cell histiocytosis, in order to avoid missing diagnosis of more aggressive types of this disorder.

## Background

Congenital self-healing reticulohistiocytosis (CSHRH), also known as Hashimoto-Pritzker disease, is a rare benign type of Langerhans cell histiocytosis (LCH) described in 1973 [1, 2].

CSHRH manifests generally at birth or during the neonatal period with multiple red-brown papules, nodules, or vesicles and spontaneous regression within a few months. Solitary lesions are also reported [3]. In addition, it is characterized by lack of systemic involvement. Nevertheless, long-term follow-up is recommended due to the unpredictable course of Langerhans cell histiocytosis [4–6].

We report a case of a newborn with cutaneous and oral mucosa involvement. In addition, a review of the literature was perfomed on Pubmed using the following mesh terms: “Congenital self-healing reticulohistiocytosis”, “congenital self-healing Langerhans cell histiocytosis” and “Hashimoto-Pritzker disease”.

## Case presentation

**Fig. 1:**
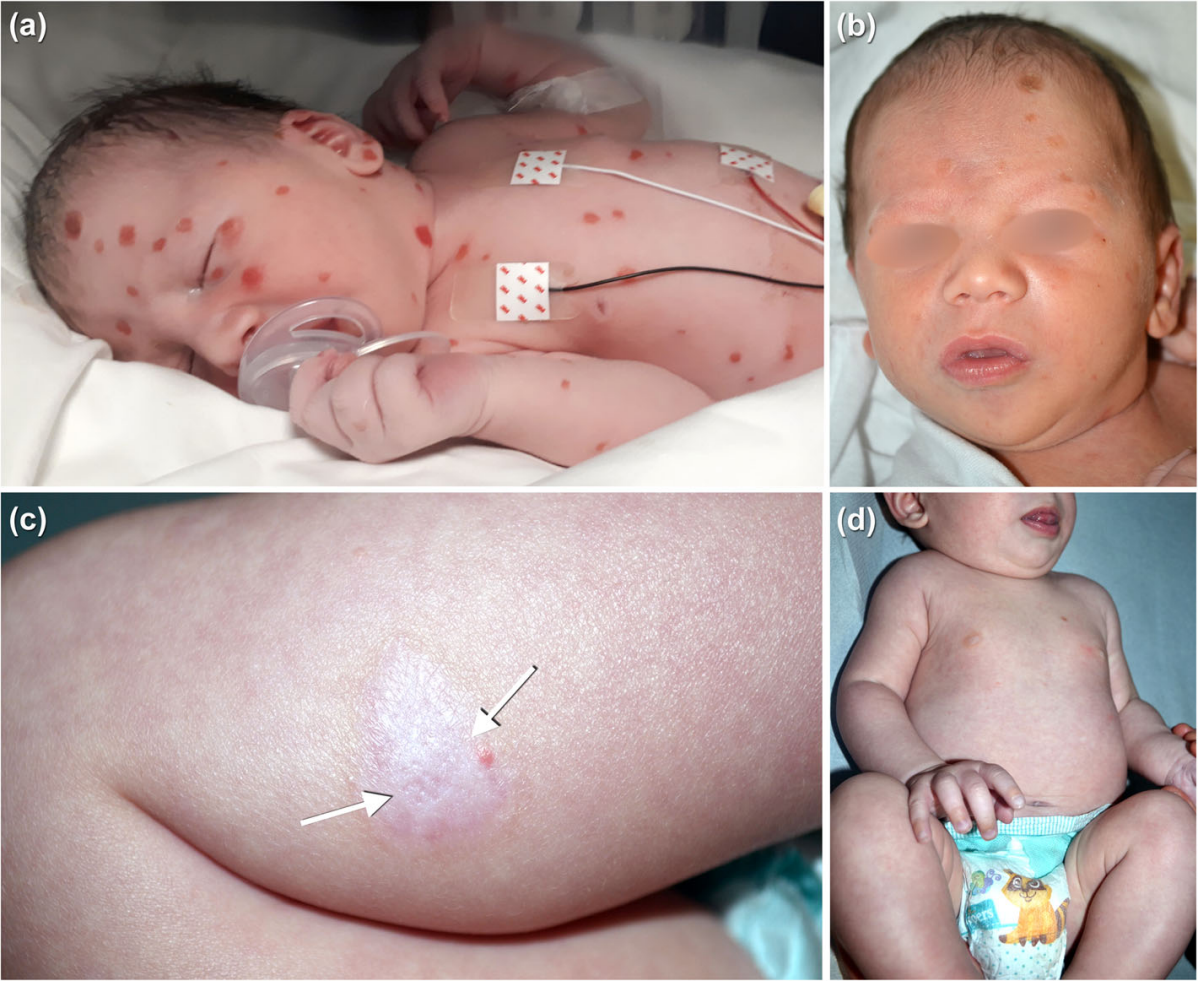
Clinical features of the patient. Round shaped erosions diffuse on the body (**A**); small erosion on the right side of the of the tongue (**B**); hypopigmented and slightly atrophic scar on the right lower leg (**C**); complete healing of the lesions at 5 months of age(**D**)

A male full term neonate, born spontaneously after an uncomplicated pregnancy from non-consanguineous parents, presented at birth multiple polymorphic cutaneous manifestations on the trunk, limbs, and head: vesicles, blisters, pustules, erythematous and exudative lesions (Fig. 1A). In addition, small erosions were present on the tongue (Fig. 1B). The baby was otherwise healthy, with appropriate gestational age (AGA), regular cardiovascular adaptation, normal tone and neonatal reflexes. He was afebrile with stable vital signs and the remainder of his physical examination was normal; no lymphadenopathy or organomegaly was noted. No family history for blistering diseases.

Congenital infections were ruled out through bacterial cultures and maternal serology for TORCH complex (Toxoplasma, Rubella, Citomegalovirus, Herpes virus, *syphilis, varicella-zoster, parvovirus B19*,). Patient’s serology for TORCH group infections, Epstein-Barr virus, Coxsackie virus A and B and Adenovirus was negative. Complete blood-cell count, kidney and liver function tests, clotting tests, and C-reactive protein were normal. Polymerase chain reaction for Herpes Simplex Virus 1/2, Human Herpes Virus 6 and Coxsackie virus on vesicular content was negative.

The mother was tested for anti-SSA and anti-SSB, and neonatal lupus was excluded.

On the fifth day of life, the baby was discharged in good general conditions and referred to Dermatology Unit at Bambino Gesù Children’s Hospital for further investigation. The patient manifested atrophic and dyschromic scars on the trunk, face and limbs; small vesicles and erythematous papules were present on the face and scalp (Fig. 1C).

**Fig. 2:**
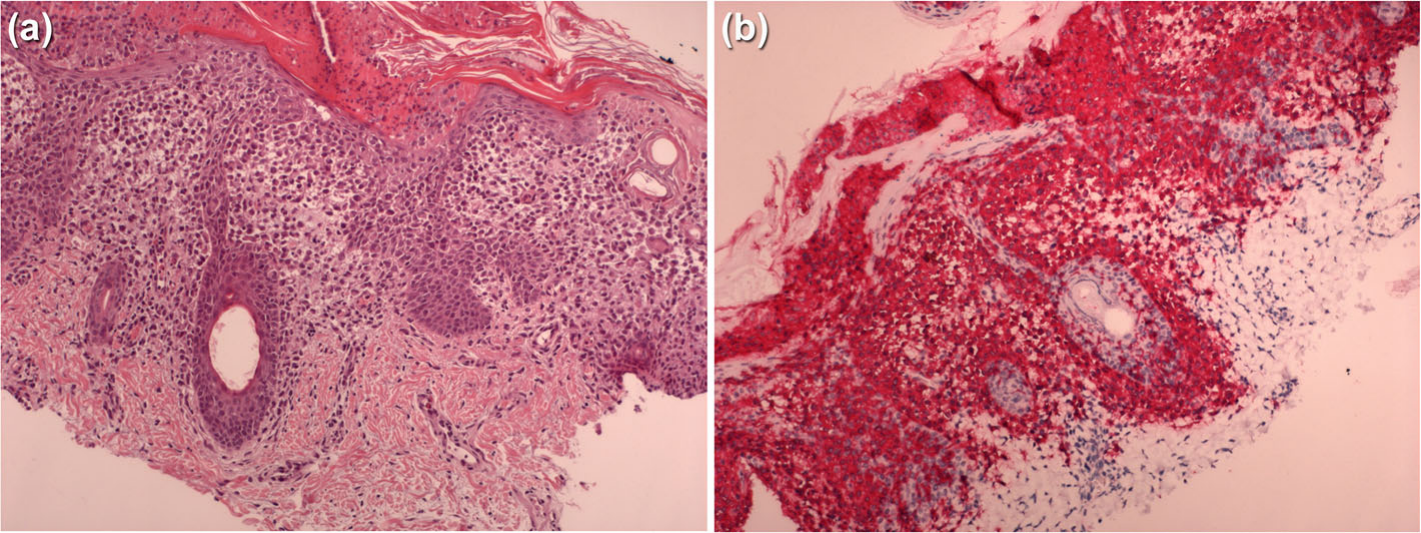
Skin biopsy shows dermal infiltrate with epidermotropism (E&E 10X) (**A**); CD1a immunostain displays positive Langherans cells (IHC 10X) (**B**)

A skin biopsy was performed. Histopathological examination showed a dense infiltrate of large histiocytic cells, with pale cytoplasm and reniform nucleus, filling the papillary dermis and infiltrating the epidermis (Fig. 2A). The histiocytes stained positive for CD1a (Fig. 2B), Langherin (CD207), S100 protein and e-cadherin. BRAF V600E mutation has been identified both with immunohistochemistry and polymerase chain reaction (PCR).

The baby was then evaluated together with a pediatric oncohematologist to perform a complete disease staging with imaging and laboratory studies.

Complete blood count, coagulation studies, serum urea, creatinine, bilirubin, liver enzymes and thyroid function tests were within normal limits. Abdominal ultrasound, whole body MRI and brain MRI seemed unremarkable.

A presumptive diagnosis of self-healing histiocytosis Hashimoto-Pritzker was established, due to almost complete resolution of the mucocutaneous lesions and absence of extracutaneous involvement. Topical mometasone fuorate ointment was initiated and a follow-up was planned.

At 2 months of age, all lesions disappeared, with some residual scars. At 5 months follow-up the patient is healthy (Fig. 1D).

## Discussion and conclusions

LCH is a rare clonal disease of the monocyte-macrophage system characterized by uncontrolled proliferation and accumulation of CD1a^+^/CD207^+^ dendritic cells [7]. The clinical behavior of LCH is remarkably heterogeneous and may range from isolated, self-healing skin and bone lesions to life-threatening multi-system condition.

Generally, patients with LCH can be divided into two groups based on the extent of involvement at diagnosis, namely, single-system LCH and multisystem LCH. The single-organ involvement may be unifocal or multifocal. Specific organs are considered high risk; they include the liver, spleen and hematopoietic system. Approximately 65% of patients have single-system disease [8]. Bone is the most common single-organ site (80% of cases) followed by the skin (12%) [9].

Congenital self-healing reticulohistiocytosis (CSHRH) is characterized by absence of systemic symptoms and spontaneous involution. In addition, dermatological manifestations are peculiar compared to the other LCH types. More than 100 cases have been reported in literature. This disease is probably underestimated due to the lack of extracutaneous involvement and spontaneous resolution within few weeks or months.

The most common presentation is characterized by diffuse multiple erythematous to purpuric-brown papulonodules or vesicles on the skin. In addition, cutaneous blisters and mucous erosions are very rarely reported. Indeed, although some authors have emphasized the absence of mucous membrane involvement, oral lesions have been described in five patients within the first days of disease manifestation [4, 10, 11]. Our patient presented since birth cutaneous blisters and few erosions in the oral mucosa without feeding difficulties [12]. Isolated lesions have been reported approximately in 25% of cases without preferred site; the disease onset is usually at birth, and in some cases within early childhood [13]. Few patients with CSHRH manifested at birth numerous blue-purplish and dark-red papular, nodular lesions; this presentation is considered part of the spectrum of blueberry muffin baby (BMB) [3]. Other rare and atypical cutaneous manifestations in children with CSHRH are diffuse hypopigmented flat-topped papules [14].

Differential diagnoses of CSHRH should consider: congenital or acquired infections (syphilis, impetigo, herpes, staphylococcal scaled-skin syndrome, etc.), autoimmune diseases (neonatal pemphigus or neonatal lupus erythematosus) or other haematological disorders [12].

Upon literature review, the most frequent sequela of CSHRH is post-inflammatory hyper- or hypopigmentation. Residual scars, milia, and anetoderma, especially in larger lesions are also reported [15]. Indeed, our patient presented dyschromic and atrophic scars on the trunk and right leg (Fig. 1C).

The diagnosis of LCH is based on histopathology and immunohistochemistry, which are the same in all forms of LCH. The hallmark cells stain positive for S100 protein, CD1a and Langherin (CD207), a monoclonal antibody direct against a type II transmembrane c-type lectin protein of Birbeck granules [16]. The activating BRAFV600E mutation has been recently identified as a frequent molecular genetic alteration both in systemic LCH and in CSHRH. The presence of circulating cells carrying the mutation is considered predictive of disseminate disease [17]. In our patient BRAFV600E mutation was present only on Langerhans cells of the skin.

The diagnosis of CSHRH is confirmed through clinical features, absence of extracutaneous involvement, early spontaneous healing, and histology. Moreover, relapse of cutaneous manifestation and/or subsequent visceral involvement including lungs, eyes, or bones have been described, especially in the first year of life. These data suggested that a long-term follow-up is mandatory [3, 5, 6, 18–20].

No specific treatment for congenital self-healing reticulohistiocytosis is required, apart from topical management for blisters and erosions as in our patient, or systemic antibiotics in case of bacterial superinfection [18].

In conclusion, our case confirms that congenital lesions of oral mucosa in CSHRH are possible. In our opinion this localization is probably underestimated due to fugacity of the erosions and difficulties in exploring the oral cavity in a newborn with normal feeding. Patients affected with LCH, without extracutaneous involvement who manifest a benign course require a follow-up until the first year of life because the diagnosis of CSHRH can be confirmed only a posteriori.

## Data Availability

Data are recorded in our intranet.

## Abbreviations

CSHRH: Congenital self-healing reticulohistiocytosis
LCH: Langerhans cell histiocytosis
AGA: Appropriate gestational age
TORCH complex: (Toxoplasma, Rubella, Citomegalovirus, Herpes virus, *syphilis, varicella-zoster, parvovirus B19*,)
BMB: Blueberry muffin baby
